# Honey bee colony performance and health are enhanced by apiary proximity to US Conservation Reserve Program (CRP) lands

**DOI:** 10.1038/s41598-019-41281-3

**Published:** 2019-03-20

**Authors:** Vincent A. Ricigliano, Brendon M. Mott, Patrick W. Maes, Amy S. Floyd, William Fitz, Duan C. Copeland, William G. Meikle, Kirk E. Anderson

**Affiliations:** 1 USDA-ARS Carl Hayden Bee Research Center, Tucson, AZ 85719 USA; 2 USDA-ARS, Honey Bee Breeding, Genetics, and Physiology Laboratory, Baton Rouge, LA 70820 USA; 3 Department of Entomology and Center for Insect Science, University of Arizona, Tucson, AZ 85721 USA; 4 Department of Microbiology, School of Animal & Comparative Biomedical Sciences, University of Arizona, Tucson, AZ 85721 USA

## Abstract

Honey bee colony performance and health are intimately linked to the foraging environment. Recent evidence suggests that the US Conservation Reserve Program (CRP) has a positive impact on environmental suitability for supporting honey bee apiaries. However, relatively little is known about the influence of habitat conservation efforts on honey bee colony health. Identifying specific factors that influence bee health at the colony level incorporates longitudinal monitoring of physiology across diverse environments. Using a pooled-sampling method to overcome individual variation, we monitored colony-level molecular biomarkers during critical pre- and post-winter time points. Major categories of colony health (nutrition, oxidative stress resistance, and immunity) were impacted by apiary site. In general, apiaries within foraging distance of CRP lands showed improved performance and higher gene expression of *vitellogenin* (*vg*), a nutritionally regulated protein with central storage and regulatory functions. Mirroring *vg* levels, gene transcripts encoding antioxidant enzymes and immune-related proteins were typically higher in colonies exposed to CRP environments. Our study highlights the potential of CRP lands to improve pollinator health and the utility of colony-level molecular diagnostics to assess environmental suitability for honey bees.

## Introduction

Insect pollinators are crucial for the maintenance of biodiversity^1,2^ and agricultural crop production which represents an estimated economic value of ~$15 billion annually in the United States alone^3^. Pollinator habitat suitability is influenced by many factors including biotic community structure, forage diversity and availability, and agrochemical exposure^4–11^. The United States Department of Agriculture Conservation Reserve Program (CRP) incentivizes reassignment of marginal croplands and wildlife habitats to long-term conservation efforts in exchange for annual payment to private landowners^12^. Recent analyses of land-use trends indicate that increased CRP enrollment could positively influence habitat suitability for honey bees in the Northern Great Plains, a region that harbors approximately 40% of all US bee colonies from the months of May through October^8,13^. The NGP represents one of the few places in the US where a long flowering season promotes tremendous colony growth highlighting the contribution of the floral landscape to US pollination services.

Conventional studies have monitored pollinator species diversity and population size in relation to landscape changes^14–17^. However, these approaches capture the effects of landscape alteration only after species populations have begun to decline. An alternative approach considers longitudinal changes in the physiology of individuals within a population across varying landscapes^18–20^. This allows for the identification of potential stress factors in real-time and can directly link foraging environment to population health. For example, more intensively cultivated landscapes and migratory beekeeping management conditions are significantly associated with reduced colony performance and increased levels of oxidative stress^8,21–26^.

Forage availability and nutrient balance are central to honey bee physiological processes such as brood production, oxidative stress response, immune function, host-microbe interactions, and overwintering survival^18,27–34^. Additionally, poor nutrition is correlated with a variety of sub-lethal effects including suppressed immunocompetence and increased susceptibility to pathogens and environmental xenobiotics^35–39^. A common objective of honey bee research is to effectively distill multiple variables into simplified metrics that accurately reflect colony performance under various landscape and management conditions. However, most studies aimed at understanding the relationship between bee health and landscape variation have neglected to measure group-level physiology that is characteristic of the colony superorganism. Because much of honey bee evolution has been driven by selective forces acting on the colony as opposed to the individual, it is logical to approach the colony as an adaptively organized entity analogous to a multicellular organism^40^. We therefore used a pooled sampling approach to overcome individual variation and more closely represent the average physiological status of a cohort of young bees localized to the brood nest. We exposed honey bee colonies to CRP lands or more intensively cultivated lands. We then evaluated the link between foraging environment, colony performance, and gene expression diagnostics of 50 pooled bees sampled from the center of the brood nest. This sampling approach relies on the strong association between spatial distribution of worker tasks within the colony and associated nutritional physiology^40–42^.

Our study assessed pre-winter and post-winter time points, which are critical periods in honey bee colony health. While summer colony losses are an emerging concern^43^, managed colony losses tend to occur primarily during the winter and are largely attributed to poor nutrition, queen failure, compromised immune function, increased pathogen loads, or a combination of factors^21,26,44–46^. The western honey bee is adapted to survive seasonal changes in forage quality and availability by storing simple sugars in the hive and complex nutrient stores within the bodies of long-lived workers. These workers become a nutrient storage caste referred to as diutinus bees which synthesize protein-rich food for a new cohort of brood following extended forage dearth^47^. This colony-level nutritional economy is largely contingent on the production and conservation of *vitellogenin* (*vg*), a nutritionally-regulated protein that is highly expressed during the months leading up to winter^32,39,48^. Diutinus bees accumulate increased levels of *vg*, which extends their life-span and improves their tolerance to starvation, disease, and oxidative stress^47,49^. We measured nutritional, antioxidant, and immune gene expression to evaluate the effects of CRP habitat restoration and nutritional landscape variation on honey bee colony physiology. To further explore the utility of colony-level molecular biomarkers, we measured the transcript expression of *vg-like* gene homologs implicated in life-span regulation and response to oxidative stress^50^.

## Results

### Effects of forage environment on colony performance

The current study examines colonies from apiaries with previously reported disease levels and colony performance data including colony size, brood production, pesticide analysis, and levels of Deformed Wing Virus, *Nosema* and *Varroa* infestation^18^. Adult bee population was estimated by hive weight data and the sealed brood area for each colony was estimated using digital imaging methods^51,52^. We analyzed two distinct apiaries within foraging proximity to CRP land (CRP-1 and CRP-2) and two distinct apiaries exposed more intensively cultivated land (Agriculture-1 and Agriculture-2).

**Figure 1:**
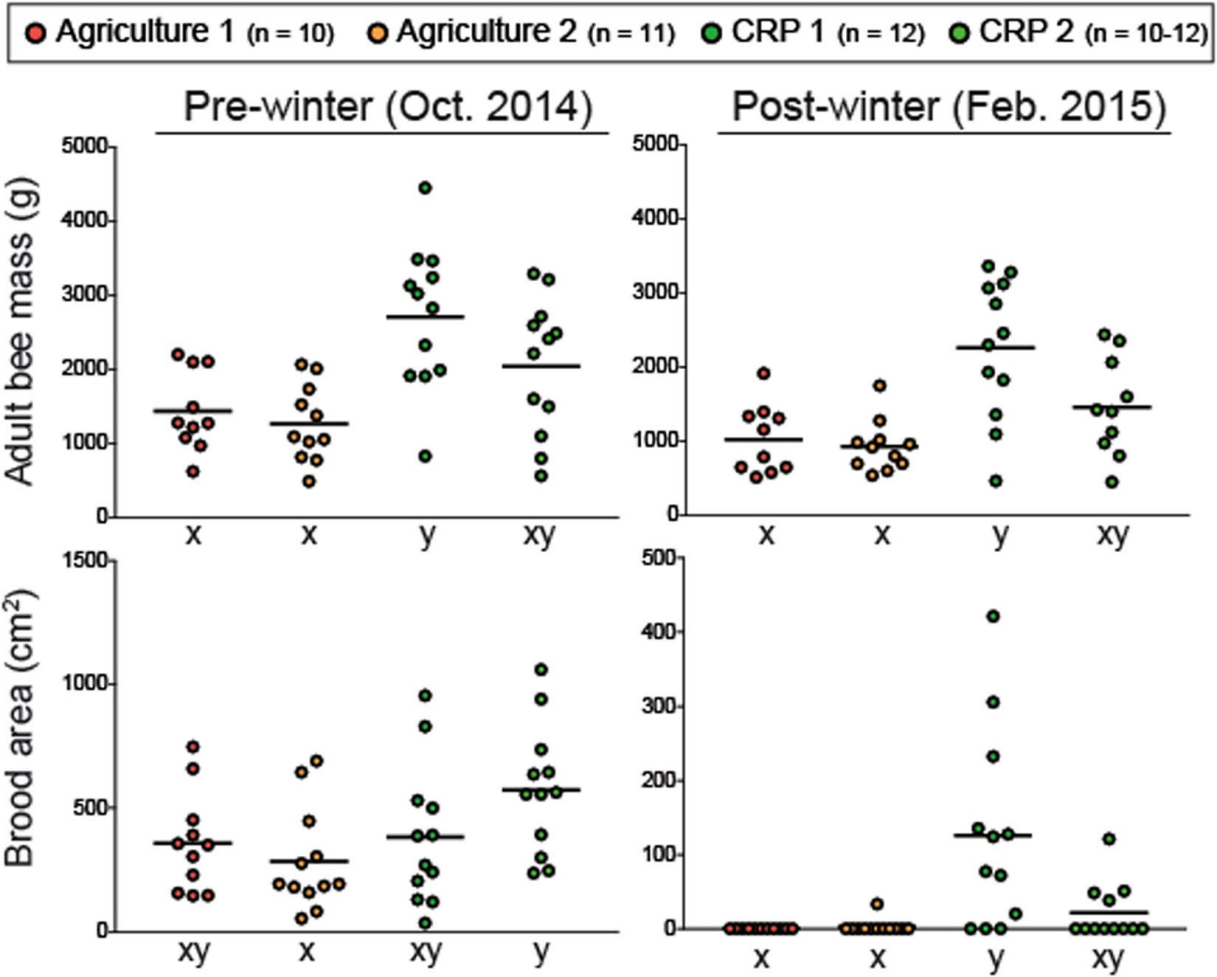
Total adult bee mass and capped brood production of colonies from different apiary sites. Black horizontal lines indicate the mean. For each performance measure and evaluation time point, different letters indicate statistically significant differences (see Supplementary Fig. S1 for detailed statistical information).

Pre-winter apiary site location significantly influenced adult bee populations (*X*^2^ = 14.89, df = 3, P = 0.002; Fig. 1a). Colonies in the CRP-1 apiary featured markedly higher pre-winter adult bee masses compared to both Agriculture sites. Colony performance at the CRP-2 site were not significantly different than Agriculture-1 or Agriculture-2 despite trending that way. Post-winter, site had a significant effect on adult bee mass (*X*^2^ = 14.86, df = 3, P = 0.002; Fig. 1b) and reflected pre-winter differences among treatment groups.

The amount of post-winter capped brood per colony was influenced by apiary location (*X*^2^ = 8.23, df = 3, P = 0.042; Fig. 1c) and showed increased pre-winter brood production at the CRP-2 apiary compared to Agriculture-2. Apiary markedly influenced post-winter capped brood production (*X*^2^ = 21.20, df = 3, P < 0.001; Fig. 1d) and colonies subjected to intensive agriclutre produced less post-winter brood overall.

*Varroa* mite, deformed wing virus (DWV) and *Nosema* levels were monitored to compare pathogen levels among sites. No significant differences were observed among sites with respect to *Varroa* mite levels, and no hives had high mite levels (Supplementary Fig. S2a,b). We previously showed that *Varroa* levels in commercially managed hives were significantly correlated with colony-level DWV transcript abundance^20^. Despite no differences in mite levels, apiary site influenced pre-winter virus levels (*X*^2^ = 9.72, df = 3, P = 0.021; Supplementary Fig. S2c) and pre-winter *Nosema* levels (*X*^2^ = 11.56, df = 3, P = 0.009; Supplementary Fig. S2e). Colonies in the Agriculture-1 site had elevated pre-winter virus levels whereas colonies in the Agriculture-2 site had elevated pre-winter *Nosema* levels. Post-winter pathogen levels were not significantly different among treatment groups, suggesting that differences in colony performance were not likely due to pathogens (Supplementary Fig. S2)

### Vitellogenin (vg) and vg-like expression

**Figure 2:**
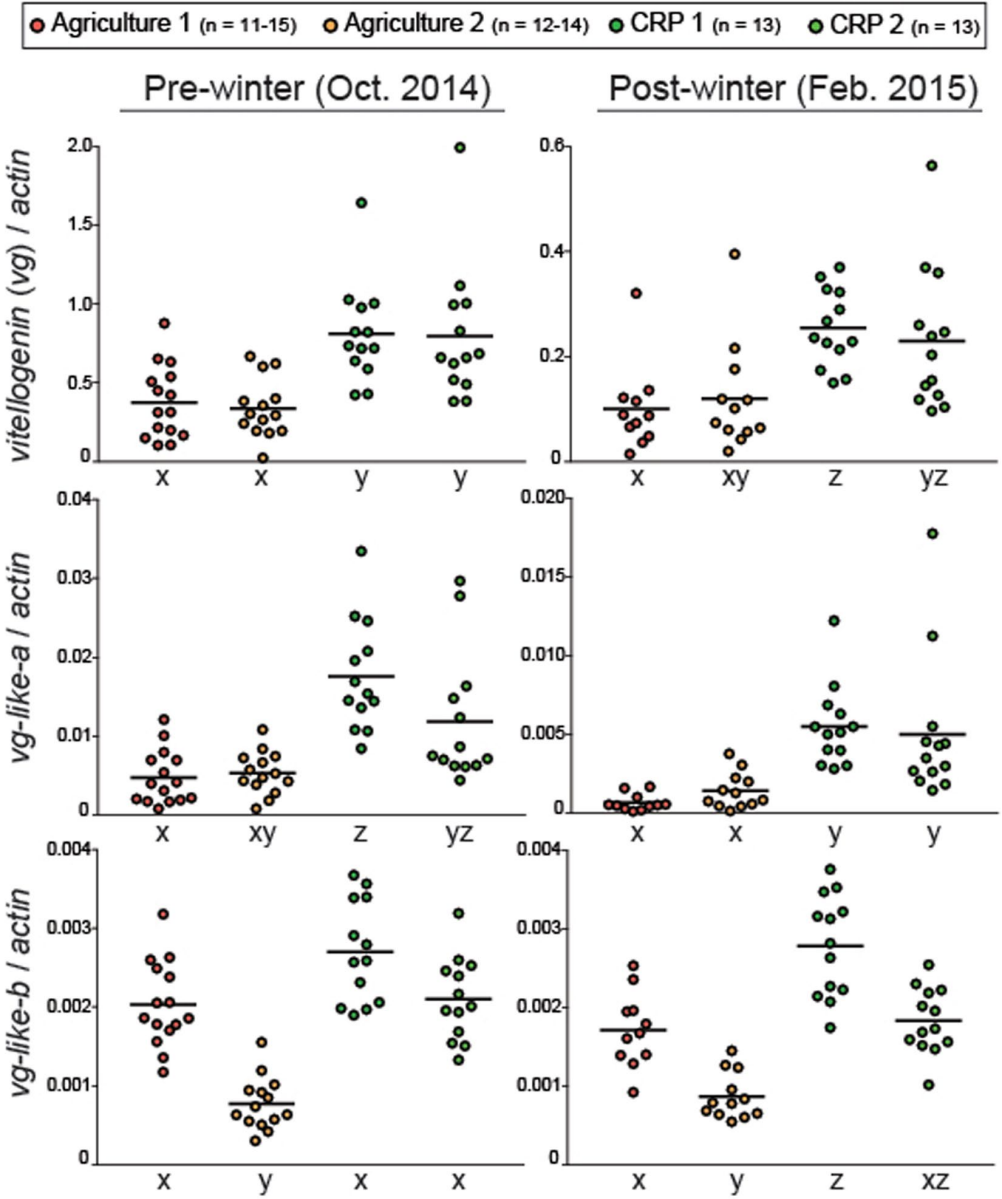
Relative colony-level expression of vitellogenin (*vg*) and *vg*-like homologs (*vg*-*like-a* and *vg-like-b*). Black horizontal lines indicate the mean. For each transcript and evaluation time point, different letters indicate statistically significant differences (see Supplementary Fig. S3 for detailed statistical information).

We profiled mRNA expression of the nutritionally regulated gene vitellogenin (*vg*) and its functional homologs (*vg-like-A* and *vg-like-B*). *vg* encodes a nutritional storage and regulatory protein that is central to honey bee processes such as brood production, aging, oxidative stress response, and overwintering. Both *vg* and the *vg-like* genes share structural and functional similarities with respect to overwintering bee phenotypes (*vg-like-A*) and oxidative stress response (*vg-like-B*)^20,50^. Apiary site significantly influenced pre-winter *vg* expression (*X*^2^ = 25.44, df = 3, P < 0.001; Fig. 2a). Pre-winter *vg* expression was approximately 2-fold higher at CRP-1 and CRP-2 relative to Agriculture-1 and Agriculture-2. Post-winter *vg* expression was significantly influenced by apiary site (*X*^2^ = 20.43, df = 3, P < 0.001; Fig. 2b). Similarly, expression was approximately 2-fold higher at CRP-1 and CRP-2 relative to Agriculture-1 and Agriculture-2.

Colony-level expression of *vg-like-A* peaks in the months leading up to winter, suggesting a potential functional role in overwintering physiology^20^. Apiary site significantly influenced pre- and post-winter *vg-like-A* levels (pre-winter: *X*^2^ = 29.67, df = 3, P < 0.001; Fig. 2c; post-winter: *X*^2^ = 32.13, df = 3, P < 0.001; Fig. 2d). Expression levels of *vg-like-A* were consistent with increased overwintering performance in colonies subjected to CRP environments.

Colony-level expression of *vg-like-B* is only modestly elevated leading up to winter^20^, but this *vg* homolog is more likely involved in oxidative stress response as evidenced in individual bees^50^. Our current results indicate that apiary site significantly influenced pre- and post-winter *vg-like-B* levels (pre-winter: *X*^2^ = 34.25, df = 3, P < 0.001 Fig. 2e; post-winter: *X*^2^ = 34.1, df = 3, P < 0.001; Fig. 2f.) Pre-winter expression of *vg-like-B* was lowest in the Agriculture-2 apiary and post-winter expression was highest in CRP-1 relative to both Agriculture apiaries.

### Colony-level *vg* expression versus total adult bee mass

**Figure 3:**
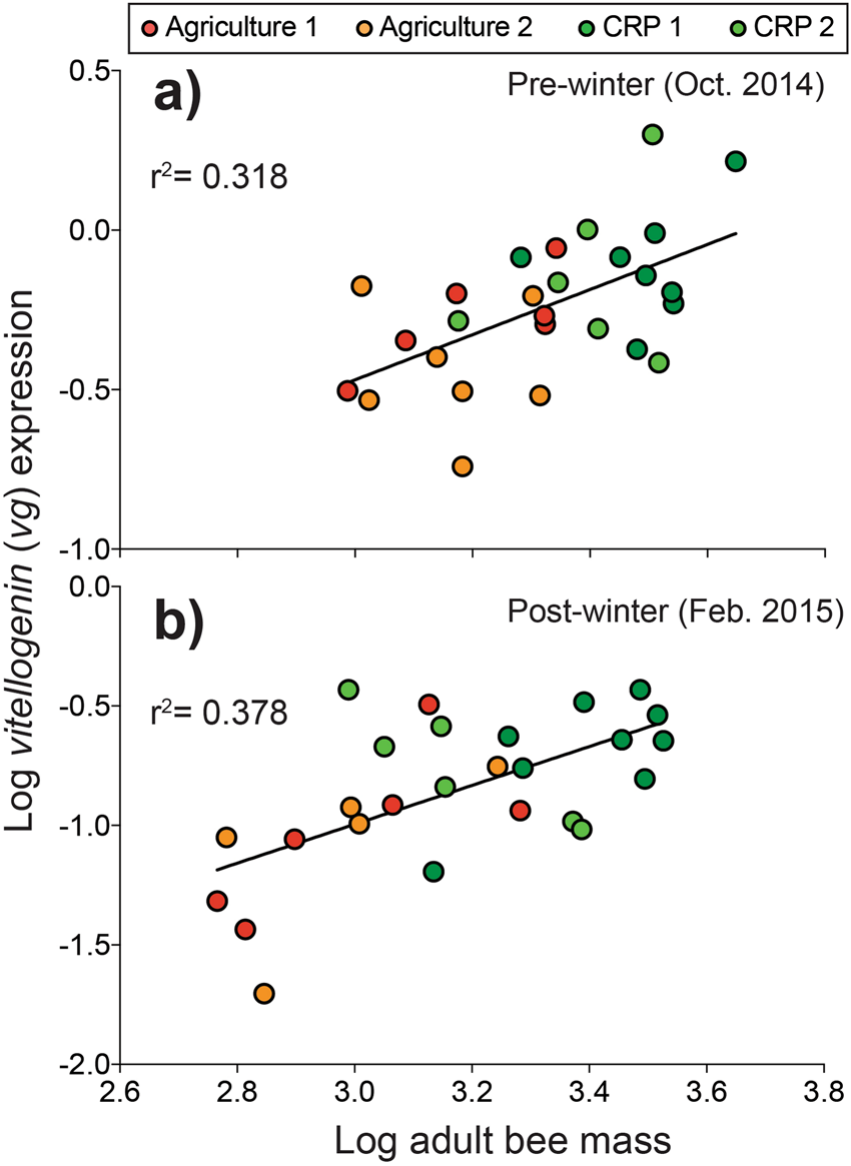
Relationship between total adult bee mass and *vitellogenin* (*vg*) expression.

Monitoring of hive weight data can provide valuable information on the interactions between colony health and the environment^51^. To test the relationship between hive weight data and colony-level molecular genetic data, we analyzed adult bee mass reported by^18^ with respect to *vg* levels from the same colonies reported here. Pre- and post-winter adult bee mass was significantly correlated with colony-level *vg* expression (pre-winter: F _1, 25_ = 11.66, P = 0.002; Fig. 3a; post-winter: F _1, 24_ = 14.57, P < 0.001; Fig. 3b). These results indicate that hive weight data and colony-level molecular diagnostics could provide complementary information in future landscape ecology studies.

### Antioxidant enzyme gene expression

The expression of antioxidant enzyme genes is associated with longevity in honey bees^53^ and is nutritionally regulated^54^. We profiled colony-level mRNA expression of the main antioxidant enzymes in honey bees (catalase, superoxide dismutase and glutathione S-transferase)^55,56^ to assess the effects of foraging environment.

**Figure 4:**
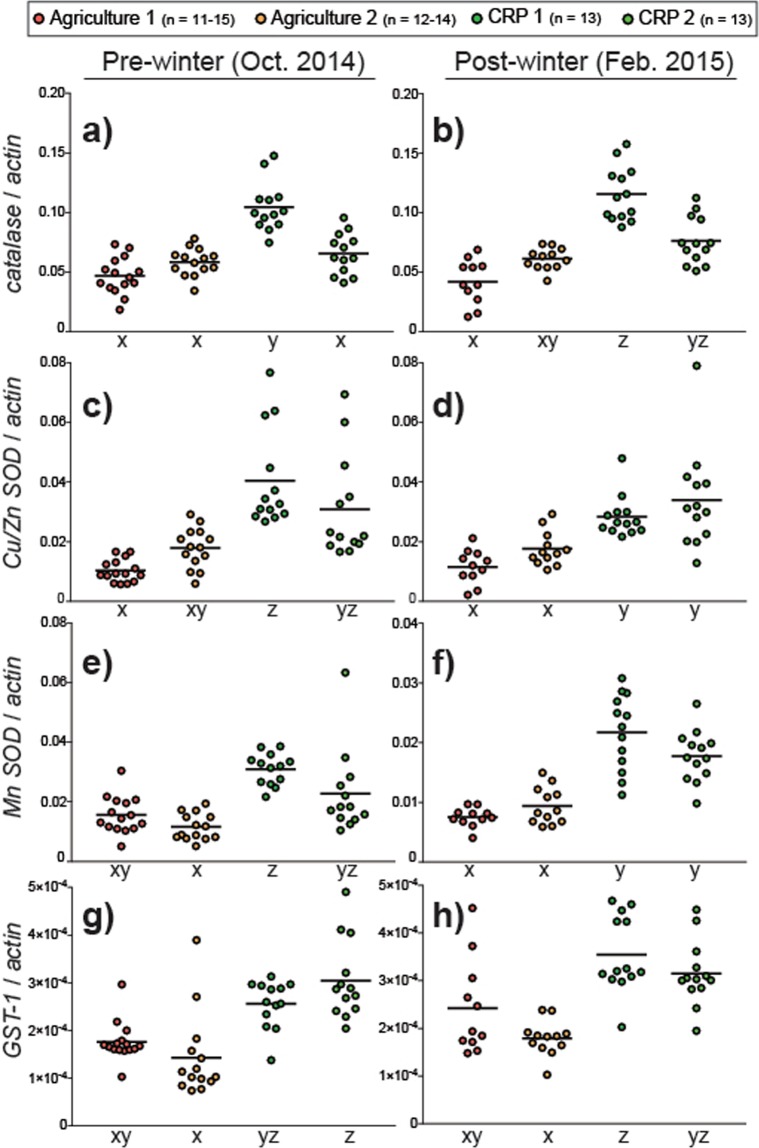
Relative colony-level expression of antioxidant enzyme transcripts. Black horizontal lines indicate the mean. For each transcript and evaluation time point, different letters indicate statistically significant differences (see Supplementary Fig. S4 for detailed statistical information).

*Catalase* detoxifies hydrogen peroxide, a reactive byproduct of normal metabolic processes, into less-reactive gaseous oxygen and water. Apiary site significantly influenced pre-winter and post-winter *catalase* expression (pre-winter: *X*^2^ = 32.54, df = 3, P < 0.001; Fig. 4a; post-winter: *X*^2^ = 33.08, df = 3, P < 0.001; Fig. 4b). Pre-winter *catalase* expression was approximately 2-fold higher in the CRP-1 apiary compared to Agriculture-1 and -2. Post-winter *catalase* expression was highest in CRP-1 and lowest in Agriculture-1.

Superoxide dismutastes (SOD) are metallo-enzymes that detoxify superoxide (O_2_^−^), one of the primary cellular ROS. The honey bee expresses a cytoplasmic SOD (*CuZn SOD*) and a mitochondrial SOD (*Mn SOD*). Pre-winter and post-winter *CuZn SOD* expression was significantly influenced by apiary site (pre-winter: *X*^2^ = 37.70, df = 3, P < 0.001; Fig. 4c; post-winter: *X*^2^ = 28.51, df = 3, P < 0.001; Fig. 4d). Pre-winter expression was highest in CRP-1 and lowest in Agriculture-1.Post-winter expression was higher at CRP sites relative to Agriculture sites.

Pre-winter and post-winter *Mn SOD* expression was significantly influenced by apiary site (pre-winter: *X*^2^ = 29.73, df = 3, P < 0.001; Fig. 4e, post-winter: *X*^2^ = 33.87, df = 3, P < 0.001; Fig. 4f). Pre-winter expression was highest in CRP-1 and lowest in Agriculture-2. Post-winter expression was higher in CRP sites relative to Agriculture sites.

Honey bee *glutathione S-transferase 1* (*Gst-1*) was shown to detoxify the prototypical xenobiotic 1-chloro-2,4-dinitrobenzene and exhibits peroxidase activity, functions that implicate it in cellular protection from ROS damage^57^. Pre-winter and post winter expression of *Gst-1* was significantly influenced by apiary site (pre-winter: *X*^2^ = 28.88, df = 3, P < 0.001; Fig. 4g; post-winter: *X*^2^ = 25.60, df = 3, P < 0.001; Fig. 4h). Pre-winter expression was highest in CRP-2 and lowest in Agriculture-2 whereas post-winter expression was highest in CRP-1 and lowest in Agriculture-2.

### Immune gene expression

**Figure 5:**
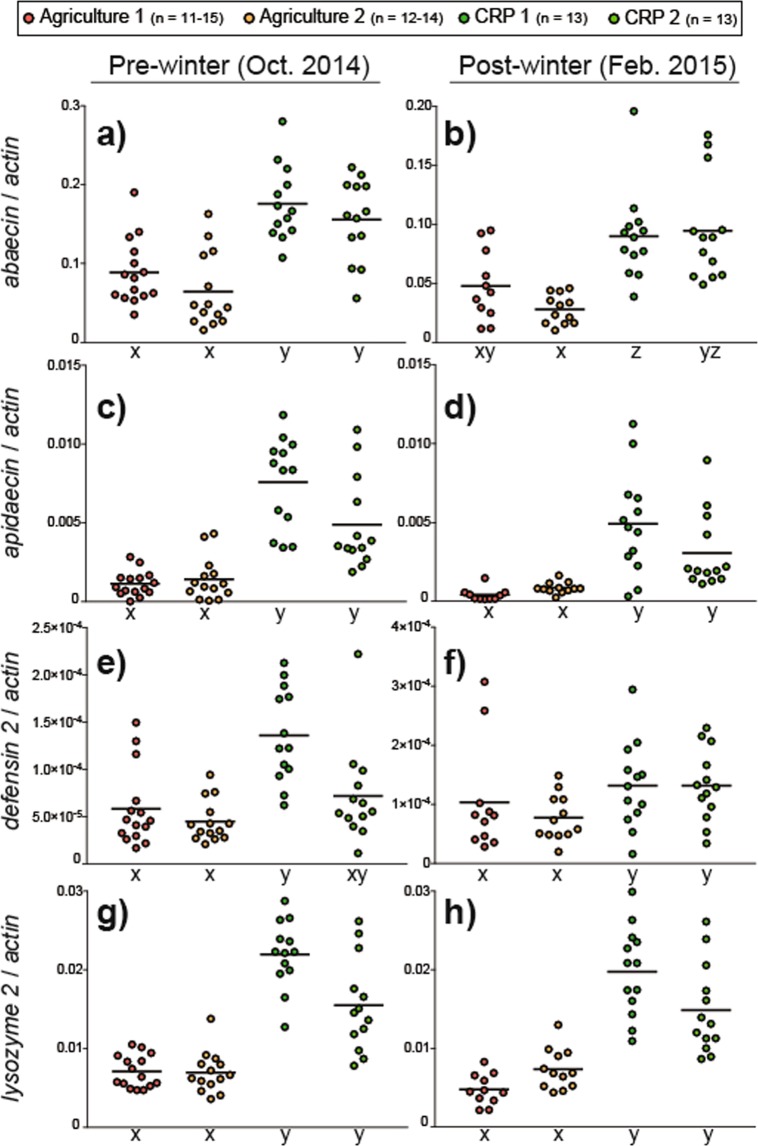
Relative colony-level expression of immune gene transcripts. Black horizontal lines indicate the mean. For each transcript and evaluation time point, different letters indicate statistically significant differences (see Supplementary Fig. S5 for detailed statistical information).

Colony-level immune status was monitored by profiling mRNA expression of antimicrobial peptides (*abaecin*, *apidaecin*, *defensin-2*) and *lysozyme-2*. These genes are constitutively expressed and are also likely nutritionally regulated^32^. Pre- and post-winter *abaecin* expression was significantly influenced by apiary site (pre-winter: *X*^2^ = 27.93, df = 3, P < 0.001; Fig. 5a; post-winter *X*^2^ = 26.51, df = 3, P < 0.001; Fig. 5b). Pre-winter abaecin expression was approximately 2-fold higher in CRP apiaries relative to Agriculture apiaries whereas post-winter expression trended towards higher expression in CRP apiaries.

Pre- and post-winter *apidaecin* levels were significantly impacted by site (pre-winter: *X*^2^ = 36.08, df = 3, P < 0.001; Fig. 5c; post-winter *X*^2^ = 29.93, df = 3, P < 0.001; Fig. 5d). At both time points, *apidaecin* expression was approximately 5- to 6-fold higher in CRP apiaries relative to Agriculture apiaries.

Pre-winter *defensin-2* levels were significantly influenced by site (*X*^2^ = 21.78, df = 3, P < 0.001; Fig. 5e) and was highest in CRP-1 and lowest in Agriculture-2. Post-winter *defensin-2* expression was not significantly influenced by apiary site (P = 0.057; Fig. 5f). Pre- and post-winter *lysozyme-2* expression were significantly influenced by site (pre-winter: *X*^2^ = 37.47, df = 3, P < 0.001; Fig. 5g; post-winter *X*^2^ = 36.16, df = 3, P < 0.001; Fig. 5h). At both time points, lysozyme-2 expression was approximately 2- to 2.5-fold higher in CRP sites compared to Agriculture sites.

## Discussion

Relative to environments with increased agricultural intensity, apiaries within foraging proximity of Conservation Reserve Program (CRP) lands exhibited greater survival potential, larger adult bee populations, increased brood production, and improved molecular biomarker profiles. Three major metrics of colony health trended towards improvement based on CRP exposure; nutrition, oxidative stress resistance, and immunity. Substantiating these metrics as critical colony health factors in this context, a previous companion study of these same colonies excluded more typical causes of colony failure including pesticides, pathogens, and parasites^18^. Our findings highlight the potential of CRP foraging environments to improve bee health and demonstrate the utility of colony-level molecular diagnostics to assess environmental suitability for honey bees. It is important to note that while these results indicate trends of improved performance and biomarker profiles, further studies are necessary to directly test the efficacy of CRP landscapes using increased apiary replication across different geographic locations.

As the target of our study, the Northern Great Plains (NGP) region of the United States harbors approximately 40% of all US honey bee colonies from the months of May through October^8^. Commercial beekeeping operations transport colonies to the NGP during the summer to produce honey and increase colony size because it contains abundant and diverse forage^23^. During the winter, colonies are typically transported from the NGP to pollinate almonds in California, or moved to southern states for queen or packaged bee production. Land-cover trends in the NGP reveal an increase in cultivated land and a reduction in the natural grasslands and wetlands that have traditionally served as forage refuge^8^. These land-use changes reduce honey bee habitat suitability and consequent colony growth and performance^8,23,24^. The Conservation Reserve Program incentivizes the removal of environmentally sensitive land from agricultural production in an effort to conserve wildlife habitats and pollinator forage^12^. However, relatively little is known about the specific effects of CRP habitat conservation programs on colony health.

Honey bee colony losses are often attributed to poor nutrition, pesticide exposure, increases in parasites/pathogens, or a combination of these factors. However, a companion study performed on the same colonies and sites reported no major differences in pesticide diversity or concentrations between apiary sites^18^, suggesting that differences in colony performance are unlikely attributable to agrochemical exposure. Similarly, levels of the main honey bee parasite (*Varroa* mite), and indicator pathogens (*Nosema* and Deformed Wing Virus) were exceedingly low in these colonies suggesting that pathogens were unlikely a major factor explaining treatment differences. A reasonable explanation for differences in colony performance are the quality of floral resources provided by CRP land, including increased quantity and diversity of pollen nutrition^18,24,58^. For this reason, our study focused on nutritionally-regulated gene expression to assess the effects of floral landscape variation at the colony level.

Relative to agriculturally intensive environments, colonies exposed to CRP lands exhibited elevated nutritional biomarkers before and after winter (Fig. 1). Floral resource availability has been linked to honey bee nutrition and health at the colony and individual levels^59,60^. A study conducted in North Dakota from 2010–2013 determined that colonies subjected to foraging environments with greater proportions of uncultivated land during the summer experienced reduced colony mortality^24^. In France, colonies placed in foraging areas with greater amounts of semi natural habitat showed increased colony-level fat body mass and *vg* expression^19^. In this study we monitored expression of *vg* and its homologs (*vg-like-A* and *vg-like-B*) as a measure of colony-level nutritional status. *Vg* is a nutritionally regulated gene that encodes a central nutritional storage protein^61,62^. Homologs of *vg* were recently identified in the genomes of all Hymenoptera and the honey bee-specific homologs exhibit functional similarities to *vg*^63^. We recently reported colony-level temporal expression patterns that were consistent with the role of *vg* and *vg-like-A* in life span regulation and winter bee phenotypes^20^. Here, we show that colonies within foraging distance to designated CRP land showed elevated levels of *vg* and *vg-like* expression, biomarkers that are likely indicative of an improved nutritional state (Fig. 2). Our findings suggest that similar to *vg*, *vg-like* genes show promise as colony-level biomarkers, which may provide improved resolution when comparing colony physiology across diverse environments (Fig. 3).

Colonies exposed to CRP lands trended towards higher levels of antioxidant gene expression, suggesting an improved capacity to mediate oxidative stress (Fig. 4). The accumulation of oxidative damage catalyzed by reactive oxygen species (ROS) is considered a universal factor in increased metabolism and the aging process, which involves damage to cellular components such as proteins and DNA by ROS, eventually leading to cellular dysfunction and death^64^. Antioxidant enzymes such as catalase, superoxide dismutase, and glutathione S-transferase ameliorate cellular damage incurred by oxidative stress. In honey bees, mRNA expression of these enzymes is nutritionally regulated and positively influenced by dietary protein levels^54^. Expression differences between apiary sites revealed a trend of higher levels of antioxidant biomarkers at sites associated with CRP foraging environments. These results mirror *vg* expression levels and suggest that improved nutritional conditions that occur with CRP exposure might augment the bee’s response to oxidative stress.

Colonies exposed to CRP lands were also associated with higher levels of immunocompetence, suggesting a capacity to counteract disease-causing microbes (Fig. 5). Honey bee immune status could be altered by the foraging landscape via nutritional quality and agrochemical exposure^7,20^. Similar to mRNA expression patterns observed for *vg* and antioxidant enzymes, colonies at CRP sites exhibited a trend of elevated immune gene expression. While increased detoxification of environmental xenobiotics could represent an energetic cost that might interfere with immunocompetence, the observed differences in gene expression are likely due to landscape nutritional quality as the number and concentration of pesticide residues detected in a concomitant study on the same colonies did not differ significantly between landscape treatments^18^. In a recent study, individual workers that consumed CRP-associated (polyfloral) diets displayed higher levels of immunocompetence compared to workers that consumed less diverse diets^31^. We hypothesize that the increased abundance and variety of floral resources in the CRP foraging environment may lead to improved immunocompetence at the colony level.

## Conclusion

Rapidly changing land use practice necessitates the identification of potential factors influencing pollinator health to inform conservation efforts. Within the context of this study, we showed that performance and biomarkers associated with adequate nutrition were positively influenced by foraging proximity to CRP land. This suggests that the removal of marginal, often environmentally sensitive land from agricultural production is a viable approach to improving bee health and pollination services. Land enrolled in the CRP supports increased floral diversity and abundance compared to more intensively cultivated land. It stands to reason that increased forage diversity and abundance may improve the occurrence of specific nutrients that are required for central honey bee physiological processes. Like all organisms, properly nourished colonies are more resistant to environmental toxins and disease. The current study revealed general trends of improved performance and health biomarkers in two distinct apiaries exposed to CRP lands relative to two apiaries exposed to intensive agriculture. Future studies employing more robust experimental designs should aim to test colony-level effects of CRP exposure across diverse environments and with increased apiary replication.

## Methods

### Honeybee Colony Management

In April of 2014, 160 colonies were established as splits from healthy parent colonies and were requeened with new Carniolan queens from a single queen supplier. Experimental colonies were moved from California to North Dakota in May 2014, where 40 colonies were placed in each of four locations. Two apiaries were surrounded by primarily non-agricultural forage: CRP-1 (46°59′44″N, 98°10′18″W), and CRP-2 (47°00′44″N, 98°05′16″W). These apiaries were typified by forage environments consisting of less than 50% agriculture (www.nass.usda.gov) and within close foraging distance (<2 km) of designated Conservation Research Program (CRP) land. Two apiaries were surrounded by more intensively cultivated lands: Agriculture-1 (46°39′47″N, 100°09′26″W) and Agriculture-2 (46°34′34″N, 100°18′01″W). These apiaries were typified by forage environments consisting primarily of sunflower and canola with alfalfa and clover blooming within forage radius of each site. See companion study by Meikle *et al*. 2017 for detailed descriptions of forage within range of each apiary site^18^.

Throughout the season, hives were treated for *Varroa* mites using standard commercial practices. In October 2014 all colonies were moved to Idaho to a common location. Hives were then overwintered indoors in climate controlled storage sheds using commercial beekeeping practices standard in the region. In February of 2015, the hives were removed from their winter storage and moved to California for almond pollination. Colonies were evaluated at a pre-winter time point (October 2014) and a post-winter time point (Feburary 2015). We re-analyzed colony performance data previously reported^18^ since the same colonies were sampled to carry out the molecular diagnostics reported here. For each colony, adult bee population was estimated using hive weight and the sealed brood area was estimated using digital imaging methods^18^.

A representative subset of 11–15 colonies per site per time point were sampled for molecular analyses. Pooled samples of brood nest bees were collected from the center of a healthy brood frame to represent a cohort of young workers based on the association between spatial variation in colony tasks and temporal polyethism^40–42^. All bees were sampled into 50 ml conical tubes, immediately frozen on dry ice, and stored at −80 °C for further processing (Supplementary Fig. S6).

### Nucleic acid extractions

Pools of 50 nurse bees were homogenized in lysis buffer (1.2M guanidine thiocyanate, 0.6M ammonium thiocyanate) using a rotary homogenizer at a volume of 0.5 ml lysis buffer per bee. One milliliter of each homogenate was added to a 2 ml bead-beating tube containing 0.2 g of 0.1 mm silica beads, immediately frozen on dry ice, and stored at −80 °C until nucleic acid extractions. Prior to extraction, the samples were thawed at 60 °C for 5 minutes, bead beaten for a total of 2 min in 30 s intervals and centrifuged to recover the supernatant. The RNA fraction was purified from 300 μl of the resulting supernatant using a GeneJet RNA Purification Kit (Thermo Fisher Scientific) according to the manufacturer’s instructions.

### Gene expression analyses

*Vitellogenin* (*vg*), *vg-like-A, vg-like-B, vg-like-C, catalase*, cytoplasmic superoxide dismutase (CuZn SOD), mitochondrial superoxide dismutase (Mn SOD), glutathione S-transferase 1 (Gst-1), *abaecin*, *apidaecin*, *defensin 2*, *lysozyme 2*, and actin mRNA levels were measured by quantitative PCR (qPCR) and cDNA template generated from the purified RNA fraction of pooled bee samples. cDNA synthesis was carried out using a RevertAid First Strand cDNA Synthesis Kit (Thermo Fisher Scientific). PCR reactions were performed in triplicate as follows: initial denaturation at 95 °C for 5 minutes; 40 cycles with denaturation at 95 °C for 15 s; and a primer-pair-specific annealing and extension temperature (Supplementary Table S1) for 30 seconds. The reactions were carried out using iTaq™ Universal SYBR® Green Supermix (Biorad) in triplicate on an CFX96™ Real-Time PCR Detection System (Biorad). To confirm the absence of contaminating genomic DNA and primer dimers in the qPCR assay, we monitored amplification and melting curves in negative controls consisting of DNase-treated total RNA without reverse transcriptase. Relative gene expression was determined based on standardized Ct values (Δ Ct)^65^ using actin as a reference gene.

### Quantification of *Varroa*, deformed wing virus, and *Nosema* levels

Fifty frozen bees from each colony were washed in alcohol, shaken through a sieve until no mites detached (at least two washes). Mites were then counted and infestation was calculated and expressed as the number of mites per bee. DWV titers were measured by qPCR using cDNA template generated from the purified RNA fraction of pooled bee homogenates^66,67^. Relative viral levels were determined based on standardized Ct values (Δ Ct)^65^ using DWV primers (Supplementary Table S1) and actin as a reference gene. *Nosema* spore counts were quantified with light microscopy (averaging paired haemocytometer counts) from a pooled sample of 15 abdomens per colony^68^.

### Statistical analyses

All analyses were conducted in JMP v11 and Prism v7. Dependent variables were evaluated for normality using fit statistics and probability plots. Variables with deviations from normality were re-evaluated after log transformation. The effects of apiary site on colony performance and gene expression were analyzed at each site and evaluation time point by Kruskal-Wallis (K-W) test and post hoc contrasts were conducted using Dunn’s test for multiple comparisons.

## Supplementary information

Supplementary information

## Data Availability

The datasets generated during and/or analyzed during the current study are available from the corresponding authors on reasonable request.
