# Supplementary material for: Honey bee colony performance and health are enhanced by apiary proximity to US Conservation Reserve Program (CRP) lands
Source: Sci Rep. 2019 Mar 20;9:4894. doi: 10.1038/s41598-019-41281-3 (PMC6426953; doi:10.1038/s41598-019-41281-3)
Supplement: Supplementary file 1 — Supplementary information [file 41598_2019_41281_MOESM1_ESM.docx]

Honey bee colony performance and health are enhanced by apiary proximity to US Conservation Reserve Program (CRP) lands

Vincent A. Ricigliano ^1,2^, Brendon M. Mott ^1^, Patrick W. Maes ^3^, Amy S. Floyd ^3^, William Fitz ^3^, Duan C. Copeland ^4^, William G. Meikle ^1^, Kirk E. Anderson ^1^

^1^ USDA-ARS Carl Hayden Bee Research Center, Tucson, AZ 85719

^2^ USDA-ARS, Honey Bee Breeding, Genetics, and Physiology Laboratory, Baton Rouge, LA 70820

^3^ Department of Entomology and Center for Insect Science, University of Arizona, Tucson, AZ 85721

^4^ Department of Microbiology, School of Animal & Comparative Biomedical Sciences; University of Arizona, Tucson, AZ, USA 85721

& Comparative Biomedical Sciences; University of Arizona, Tucson, AZ, USA 85721

Corresponding author emails: [Vincent.Ricigliano@ARS.USDA.gov](mailto:Vincent.Ricigliano@ARS.USDA.gov) and [Kirk.Anderson@ARS.USDA.GOV](mailto:Kirk.Anderson@ARS.USDA.GOV)

**Figure S1.** Summary of post hoc contrasts between performance of apiary sites using Dunn’s test for multiple comparisons

**Figure S2.** *Varroa*, deformed wing virus, and *Nosema* levels in colonies from different apiary sites.

**Figure S3.** Summary of post hoc contrasts between apiary site effects on colony-level *vitellogenin (vg)* and *vg-like* expression using Dunn’s test for multiple comparisons

**Figure S3 (continued).** Summary of post hoc contrasts between apiary site effects on colony-level *vitellogenin (vg)* and *vg-like* expression using Dunn’s test for multiple comparisons

**Figure S4.** Summary of post hoc contrasts between apiary site effects on colony-level antioxidant enzyme gene expression using Dunn’s test for multiple comparisons

**Figure S4 (continued).** Summary of post hoc contrasts between apiary site effects on colony-level antioxidant enzyme gene expression using Dunn’s test for multiple comparisons

**Figure S5.** Summary of post hoc contrasts between apiary site effects on colony-level immune gene expression using Dunn’s test for multiple comparisons

**Figure S5 (continued).** Summary of post hoc contrasts between apiary site effects on colony-level immune gene expression using Dunn’s test for multiple comparisons

**Figure S6.** Schematic overview of pooled-bee sample processing. Image was created by the authors for use in this manuscript.

**Table S1.** Primers used in this study

| **Gene**  **(accession number)** | **Forward 5’-3’** | **Reverse 5’-3’** | **Annealing temperature (°C)** | **Study** |
| --- | --- | --- | --- | --- |
| *actin*  (XM_623378) | TGCCAACACTGTCCTTTCTG | AGAATTGACCCACCAATCCA | 55.0 | Alaux et al., 2011 |
| *vitellogenin (vg)*  (AJ517411) | GTTGGAGAGCAACATGCAGA | TCGATCCATTCCTTGATGGT | 57.5 | Salmela et al., 2016 |
| *vg-like-A* (XM_001121939.3) | GTTTATGACGAAAATGGACACCT | TGAACAGTTTCCTCGTGAGTT | 57.5 | Salmela et al., 2016 |
| *vg-like-B*  (XM_006561115.1) | ATTGCAACAATGCAGTTTGAATCAT | GCAAATCAGCCACATCTTTAGGA | 57.5 | Salmela et al., 2016 |
| Deformed wing virus (DWV) | CAGTAGCTTGGGCGATTGTT | AGCTTCTGGAACGGCAGATA | 56.0 | Cox-Foster et al., 2007 |
| *abaecin*  (GB18323) | CAGCATTCGCATACGTACCA | GACCAGGAAACGTTGGAAAC | 55.0 | Evans et al., 2006 |
| *apidaecin*  *(*GB17782) | TAGTCGCGGTATTTGGGAAT | TTTCACGTGCTTCATATTCTTCA | 55.0 | Evans et al., 2006 |
| *defensin2*  *(*GB10036) | GCAACTACCGCCTTTACGTC | GGGTAACGTGCGACGTTTTA | 55.0 | Evans et al., 2006 |
| *lysozyme2*  (**GB15106**) | **CCAAATTAACAGCGCCAAGT** | **GCAATTCTTCACCCAACCAT** | 55.0 | Evans et al., 2006 |
| CuZn Sod  (NM_001178027) | TCAACTTCAAGGACCACATAGTG | ATAACACCACAAGCAAGACGAG | 60.0 | Li et al., 2014 |
| Mn Sod  (NM_001178048) | GTCGCCAAAGGTGATGTCAATAC | CGTCTGGTTTACCGCCATTTG | 60.0 | Li et al., 2014 |
| *catalase*  (NM_001178069) | TTCTACTGTGGGTGGCGAAAG | GTGTGTTGTTACCGACCAAATCC | 60.0 | Li et al., 2014 |
| Gst-1  (NM_001178028) | AGGAGAGGTGTGGAGAGATAGTG | CGCAAATGGTCGTGTGGATG | 60.0 | Li et al., 2014 |
|  |  |  |  |  |
